# Comparative genomic analyses of multiple backcross mouse populations suggest *SGCG* as a novel potential obesity-modifier gene

**DOI:** 10.1093/hmg/ddac150

**Published:** 2022-07-07

**Authors:** Tanja Kuhn, Katharina Kaiser, Sandra Lebek, Delsi Altenhofen, Birgit Knebel, Ralf Herwig, Axel Rasche, Angela Pelligra, Sarah Görigk, Jenny Minh-An Khuong, Heike Vogel, Annette Schürmann, Matthias Blüher, Alexandra Chadt, Hadi Al-Hasani

**Affiliations:** Medical Faculty, Institute for Clinical Biochemistry and Pathobiochemistry, German Diabetes Center (DDZ), Heinrich Heine University, Duesseldorf D-40225, Germany; German Center for Diabetes Research (DZD), Partner Düsseldorf / Partner Potsdam, Munich-Neuherberg, Germany; Medical Faculty, Institute for Clinical Biochemistry and Pathobiochemistry, German Diabetes Center (DDZ), Heinrich Heine University, Duesseldorf D-40225, Germany; German Center for Diabetes Research (DZD), Partner Düsseldorf / Partner Potsdam, Munich-Neuherberg, Germany; Medical Faculty, Institute for Clinical Biochemistry and Pathobiochemistry, German Diabetes Center (DDZ), Heinrich Heine University, Duesseldorf D-40225, Germany; German Center for Diabetes Research (DZD), Partner Düsseldorf / Partner Potsdam, Munich-Neuherberg, Germany; Medical Faculty, Institute for Clinical Biochemistry and Pathobiochemistry, German Diabetes Center (DDZ), Heinrich Heine University, Duesseldorf D-40225, Germany; German Center for Diabetes Research (DZD), Partner Düsseldorf / Partner Potsdam, Munich-Neuherberg, Germany; Medical Faculty, Institute for Clinical Biochemistry and Pathobiochemistry, German Diabetes Center (DDZ), Heinrich Heine University, Duesseldorf D-40225, Germany; German Center for Diabetes Research (DZD), Partner Düsseldorf / Partner Potsdam, Munich-Neuherberg, Germany; Department of Computational Molecular Biology, Max Planck Institute for Molecular Genetics, Berlin D-14195, Germany; Department of Computational Molecular Biology, Max Planck Institute for Molecular Genetics, Berlin D-14195, Germany; Medical Faculty, Institute for Clinical Biochemistry and Pathobiochemistry, German Diabetes Center (DDZ), Heinrich Heine University, Duesseldorf D-40225, Germany; German Center for Diabetes Research (DZD), Partner Düsseldorf / Partner Potsdam, Munich-Neuherberg, Germany; Medical Faculty, Institute for Clinical Biochemistry and Pathobiochemistry, German Diabetes Center (DDZ), Heinrich Heine University, Duesseldorf D-40225, Germany; German Center for Diabetes Research (DZD), Partner Düsseldorf / Partner Potsdam, Munich-Neuherberg, Germany; Medical Faculty, Institute for Clinical Biochemistry and Pathobiochemistry, German Diabetes Center (DDZ), Heinrich Heine University, Duesseldorf D-40225, Germany; German Center for Diabetes Research (DZD), Partner Düsseldorf / Partner Potsdam, Munich-Neuherberg, Germany; German Center for Diabetes Research (DZD), Partner Düsseldorf / Partner Potsdam, Munich-Neuherberg, Germany; Department of Experimental Diabetology, German Institute of Human Nutrition Potsdam-Rehbruecke, Nuthetal D-14558, Germany; German Center for Diabetes Research (DZD), Partner Düsseldorf / Partner Potsdam, Munich-Neuherberg, Germany; Department of Experimental Diabetology, German Institute of Human Nutrition Potsdam-Rehbruecke, Nuthetal D-14558, Germany; Helmholtz Institute for Metabolic, Obesity and Vascular Research (HI-MAG) of the Helmholtz Zentrum München at the University of Leipzig and University Hospital Leipzig, Leipzig D-04103, Germany; Medical Faculty, Institute for Clinical Biochemistry and Pathobiochemistry, German Diabetes Center (DDZ), Heinrich Heine University, Duesseldorf D-40225, Germany; German Center for Diabetes Research (DZD), Partner Düsseldorf / Partner Potsdam, Munich-Neuherberg, Germany; Medical Faculty, Institute for Clinical Biochemistry and Pathobiochemistry, German Diabetes Center (DDZ), Heinrich Heine University, Duesseldorf D-40225, Germany; German Center for Diabetes Research (DZD), Partner Düsseldorf / Partner Potsdam, Munich-Neuherberg, Germany

## Abstract

To nominate novel disease genes for obesity and type 2 diabetes (T2D), we recently generated two mouse backcross populations of the T2D-susceptible New Zealand Obese (NZO/HI) mouse strain and two genetically different, lean and T2D-resistant strains, 129P2/OlaHsd and C3HeB/FeJ. Comparative linkage analysis of our two female backcross populations identified seven novel body fat-associated quantitative trait loci (QTL). Only the locus *Nbw14* (NZO body weight on chromosome 14) showed linkage to obesity-related traits in both backcross populations, indicating that the causal gene variant is likely specific for the NZO strain as NZO allele carriers in both crosses displayed elevated body weight and fat mass. To identify candidate genes for *Nbw14*, we used a combined approach of gene expression and haplotype analysis to filter for NZO-specific gene variants in gonadal white adipose tissue, defined as the main QTL-target tissue. Only two genes, *Arl11* and *Sgcg*, fulfilled our candidate criteria. In addition, expression QTL analysis revealed *cis*-signals for both genes within the *Nbw14* locus. Moreover, retroviral overexpression of *Sgcg* in 3T3-L1 adipocytes resulted in increased insulin-stimulated glucose uptake. In humans, mRNA levels of *SGCG* correlated with body mass index and body fat mass exclusively in diabetic subjects, suggesting that *SGCG* may present a novel marker for metabolically unhealthy obesity. In conclusion, our comparative-cross analysis could substantially improve the mapping resolution of the obesity locus *Nbw14.* Future studies will throw light on the mechanism by which *Sgcg* may protect from the development of obesity.

## Introduction

The pathogenesis of type 2 diabetes (T2D), which is characterized by chronically elevated blood glucose levels, is driven by genetic factors and their interaction with the environment (1,2). Approximately 90% of all patients with T2D are obese (3), underscoring obesity as the main risk factor in the disease development. Until today, the majority of genetic factors that predispose individuals to the development of obesity and T2D remain unknown (4,5). Genome-wide association studies (gWAS), in which clinical parameter are correlated with hundreds of thousands single nucleotide polymorphisms (SNPs), have nominated several hundred risk gene variants that are available in public databases (6). However, as the majority of the SNPs map into non-coding regions, functional evidence connecting genetic alterations with pathogenic processes of T2D is lacking for most of the genes. Therefore, mouse models that can be used for the engineering of gene mutations with well-established molecular genetic tools remain essential to study the molecular basis of T2D (7).

Numerous mouse strains are available that show a wide range of T2D-related phenotypes, similar as observed in the human population (7,8). By taking advantage of their divergent phenotypes, linkage studies on segregating outcross populations in combination with additional strategies, such as gene expression and bioinformatic analyses of the critical regions, have identified several novel T2D-modifier genes (9,10). However, linkage studies on a single population consisting of two inbred strains are limited; it will only survey loci that are genetically distinct between the two strains. Therefore, the comparative analysis of multiple inbred populations can substantially improve the mapping resolution of candidate disease genes (11–14).

The polygenetic New Zealand Obese (NZO) mouse strain has been frequently used as a model for spontaneous polygenetic obesity and T2D. Both genders develop impaired glucose tolerance and obesity in response to a high fat diet (15); however, subsequent T2D progressing into pancreatic islet failure is limited to the males, whereas the females benefit from the protective influence of the hormone oestrogen (16,17).

Several researchers (18–24), including ourselves (11–13,25,26), have used the NZO strain in genome-wide linkage studies attempting to uncover the gene variants that may drive the high susceptibility for obesity and T2D. All these studies were successful in identifying novel genomic regions, designated quantitative trait loci (QTL), associated with T2D-related phenotypes. However, due to huge nomination of candidate genes, the causal gene variants are still unknown for most of the identified QTL. Until today, only a few genes could have been functionally linked to the development of obesity and T2D in the obese NZO strain, including *Ifgga2*, *Abcc8* and *Pcpt* (12,22,27).

To uncover novel gene variants that drive the prevalence for obesity and T2D in humans, we recently conducted crossbreeding experiments using the obese and T2D-susceptible NZO, and the two lean mouse strains, C3H/FeJ and 129P2/OlaHsd (11). Comparative linkage analysis of our two outcross populations and subsequent QTL mapping revealed two novel obesity-related gene variants that may influence adipose tissue function. For one of the two genes, *sarcoglycan gamma* (*Sgcg)*, we provide functional evidence in glucose uptake in 3T3-L1 adipocytes.

## Results

### Diverging prevalence for obesity and insulin-resistance in the parental mouse strains

**Figure 1:**
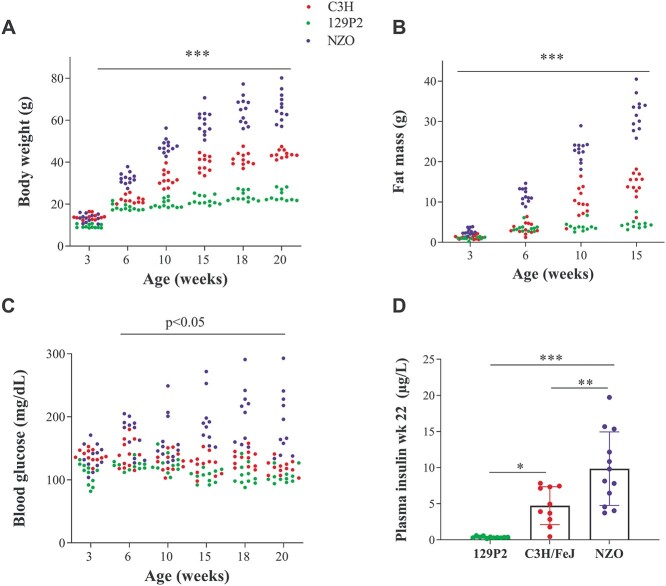
Metabolic characterization of females from parental mouse strains 129P2, C3H and NZO. Development of body weight (**A**) and blood glucose (**C**) was monitored at weeks 3, 6, 10, 15, 18 and 20 weeks of age, whereas fat mass (**B**) was measured at weeks 3, 6, 10 and 15. Six-hours-fasting plasma insulin levels (**D**) were measured at week 22 by ELISA. Dots represent single female animals (129P2: n = 11–12; C3H: n = 10–12; NZO: n = 12). Statistical differences between strains were calculated by 2-way (A-C) or one-way analysis of variance (D) followed by post hoc Bonferroni test, ^*^P < 0.05, ^*^^*^P < 0.01, ^*^^*^^*^P < 0.001 by comparison to NZO unless otherwise stated.

To validate the diverse metabolic features from our parental strains we monitored the development of body weight, body composition, blood glucose, and plasma insulin levels in female NZO, C3H and 129P2 mice. After weaning at 3 weeks of age, body weight was comparable between NZO and C3H, but significantly lower in 129P2 mice (NZO 13.4 ± 0.5 g, C3H 14.0 ± 0.4 g, 129P2 9.5 ± 0.2 g). As expected, during high-fat diet (HFD)-intervention NZO mice exhibited markedly higher body weight compared to the other two strains. At the age of 20 weeks, NZO mice gained 22 g and 42 g more than C3H and 129P2, respectively (NZO 66.4 ± 2 g, C3H 43.9 ± 0.5 g, 129P2 24.1 ± 0.8 g; Fig. 1A). These differences in body weight were mainly attributed to substantial differences in fat mass (week 15 of age: NZO 32.2 ± 1.6 g, C3H 13.9 ± 1 g, 129P2 5.76 ± 1.1 g; Fig. 1B), whereas lean mass was only slightly different between the strains (week 15 of age: NZO 25.7 ± 0.4 g, C3H 22.4 ± 0.7 g, 129P2 17.3 ± 0.7 g). Moreover, upon HFD NZO mice exhibited significantly higher blood glucose levels compared to the two lean strains. Maximal differences in glycaemia were observed at 18 week of age, when NZO mice exhibited 99 mg/dl and 123 mg/dl higher blood glucose levels compared to C3H and 129P2 mice, respectively (NZO 231 ± 22 mg/dl, C3H 132 ± 4 mg/dl, 129P2 108 ± 5 mg/dl; Fig. 1C). In addition, 6-hour fasting plasma insulin levels at the age of 22 weeks were 2-fold and 10-fold higher in NZO compared to C3H and 129P2 mice, respectively (NZO 9.9 ± 1.4 μg/L, C3H 5.1 ± 0.9 μg/L, 129P2 0.7 ± 0.3 μg/L; Fig. 1D).

### Genome-wide linkage analysis on both N_2_ populations revealed seven novel obesity QTL

**Figure 2:**
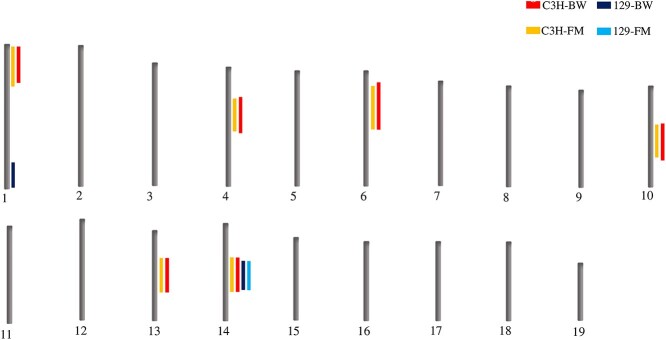
Genome-wide linkage map of QTL for obesity-related traits in female mice derived from N_2_(NZOxC3H) and N_2_(NZOx129P2) outcross populations. Female NZO and male 129P2 or C3H mice, respectively, were used to generate the F_1_ generations, and male F_1_ offspring were subsequently backcrossed with NZO females to generate two backcross populations (N_2_(NZOxC3H) and N_2_(NZOx129P2)). The backcross populations were metabolically phenotyped, and genotyped for 105 N_2_(NZOxC3H) and 110 N_2_(NZOx129P2) informative SNP markers. Linkage analysis of the female backcross mice (n = 307–309) was conducted using the R-package as described in methods. Further information for each QTL is shown in Table 1. BW, body weight; FM, fat mass.

NZO females were bred either with C3H or 129P2 males to generate two F1 (F_1_(NZOxC3H) and F_1_(NZOx129P2)) generations. Subsequently, males from the F1 generations were bred with NZO females to generate two N_2_ populations. In total, 307 females from the N_2_(NZOx129P2) population and 310 females from the N_2_(NZOxC3H) population were metabolically phenotyped for T2D-associated traits. In this study, we present all significant obesity QTL, meaning loci that are linked to body weight and/or fat mass development, identified in our backcross females. In total, we identified seven novel obesity QTL (Fig. 2 and Table 1). Five QTL were identified exclusively in the N_2_(NZOxC3H) population (*Nbw1p*, *Nbw4p*, *Nbw6*, *Nbw10* and *Nbw13*), whereas only one QTL on distal chromosome 1 (*Nbw1d*) was unique for the N_2_(NZOx129P2) cross. The QTL on proximal chromosome 14, designated *Nbw14* (NZO body weight on chromosome 14) was the only QTL shared by both N_2_(NZOxC3H) and N_2_(NZOx129P2) crosses. Details on the seven different QTL, such as the confidence interval and the allelic effects are displayed in Table 1.

**Table 1:** Summary of the major obesity QTL with significance for several weeks from female mice from both N_2_(NZOxC3H and NZOx129P2) populations

Name	Chr	Traits	Peak-Pos (cM)	95% CI (cM)	Closest SNP-marker (Mbp)	Max. LOD	Signif. weeks (max. effect)	Mean NZO/NZO	Mean NZO/C3H or NZO/129P2	Cross
*Nbw1p*	1	BW	9	0–20	46.3	4.0	10	45.2 g	42.4 g	NZOxC3H
		LM	18	2–30	46.9	3.0	10	23.1 g	22.3 g	NZOxC3H
		FM	8	0–22	27.3	3.6	6–10 (10)	20.3 g	18.3 g	NZOxC3H
*Nbw1d*	1	BW	19	16–20	182.9	3.2	15–20 (20)	53.0 g	49.1 g	NZOX129P2
		BG	19	13–20	182.9	3.5	20	147 mg/dl	134 mg/dl	NZOX129P2
*Nbw4p*	4	BW	15	7–26	53	6.93	10–20 (19)	62.1 g	56 g	NZOxC3H
	4	FM	15	8–25	53	7.54	10–15 (15)	28.7 g	24.7 g	NZOxC3H
*Nbw6*	6	BW	20	9–52	64.2	3.9	6–20 (6)	31.7 g	30 g	NZOxC3H
		FM	20	5–50	64.2	4.4	6–15 (6)	10.5 g	9.3 g	NZOxC3H
*Nbw10*	10	BW	7.2	5–11	89.1	9.3	6–20 (6)	32.1 g	29.5 g	NZOxC3H
		FM	7.2	3–10	89.1	6.3	6–20 (10)	20.6 g	17.8 g	NZOxC3H
*Nbw13*	13	BW	29.04	0–29	74.4	4.2	6–10 (6)	31.8 g	30 g	NZOxC3H
		FM	29	0–29	74.3	3.9	6–10 (6)	10.4 g	9.3 g	NZOxC3H
*Nbw14*	14	BW	23	17–30	63.6	5.2	6–20 (6)	31.8 g	29.8 g	NZOxC3H
		FM	23	17–32	63.5	6.3	6–15 (6)	10.6 g	9.2 g	NZOxC3H
		BW	32	25–40	65.4	4.4	15–20 (20)	53.8 g	49.2 g	NZOX129P2
		FM	33	26–40	65.4	3.8	10–15 (15)	22.7 g	20.3 g	NZOX129P2
		BG	29	22–37	65.4	3.1	20	148 mg/dl	136 mg/dl	NZOX129P2

LOD scores, peak positions, and 95% confidence intervals (Bayesian method) were calculated by R/qtl software. NZOxC3H: n = 310, NZOx129P2: n = 307 females. BG, blood glucose; BW, body weight; Chr, chromosome; CI, confidence interval; FM, fat mass; LM, lean mass; LOD, logarithm of the odds; Pos, Position; Signif, significant.

### *Nbw14* represents the only obesity QTL that appeared in both crossbreedings

**Figure 3:**
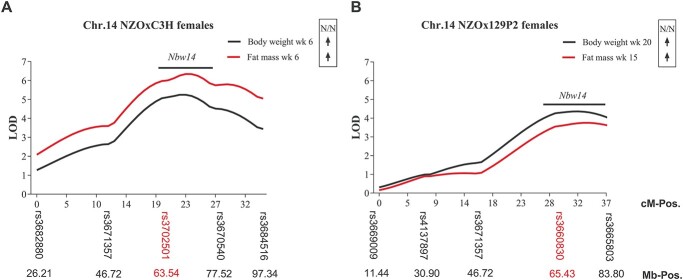
Genetic linkage of body weight and fat mass to loci on Chr.14 in outcross populations of N_2_(NZOxC3H) and N_2_(NZOx129P2) female mice. Chr.14 revealed significant linkage with body weight and fat mass in both, N_2_(NZOxC3H) (**A**) and N_2_(NZOx129P2) (**B**) females. The SNP-markers that were used for genotyping with the corresponding Mb-position are plotted below. The SNP markers in closest proximity to the QTL peak position are marked in red. The genotypic effects are indicated with the arrows. LOD scores were calculated using R/qtl software as described in methods. LOD, logarithm of the odds; N, NZO; wk, week.

Out of our seven obesity QTL, only one of the loci showed overlap between the two crosses. *Nbw14* revealed significant linkage with body weight (LOD 5.2 at 23 cM in N_2_(NZOxC3H) and LOD 4.4 at 32 cM in N_2_(NZOx129P2)) and fat mass (LOD 6.3 at 23 cM in N_2_(NZOxC3H) and LOD 3.7 at 33 cM in N_2_(NZOx129P2)) in both backcross populations. In addition, *Nbw14* was further associated with blood glucose levels in N_2_(NZOx129P2) mice (LOD 3.1 at 29 cM, closest SNP marker at 65.4 Mb). In both crosses, the SNP marker associated with the highest LOD score for body weight and fat mass appeared almost at identical position (rs3702501 at 63.5 Mb in N_2_(NZOxC3H), Fig. 3A; rs3660830 at 65.4 Mb N_2_(NZOx129P2), Fig. 3B).

### NZO-allele for *Nbw14* is associated with increased body weight and fat mass in both crossbreedings

**Figure 4:**
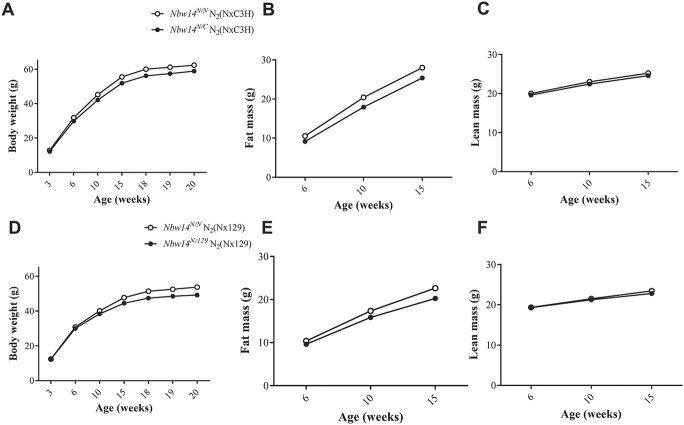
Quantitative effect of *Nbw14* in N_2_(NZOxC3H) and N_2_(NZOx129P2) mice. Mean values for body weight (± SEM) (**A**), fat mass (**B**) and lean mass (**C**) for homozygous NZO and heterozygous allele carriers for *Nbw14* the N_2_(NZOxC3H) females. Mean values for body weight (± SEM) (**D**), fat mass (**E**) and lean mass (**F**) for homozygous NZO and heterozygous allele carriers for *Nbw14* from N_2_(NZOx129P2) females. *Nbw14^NZO/NZO^* (N_2_(NZOxC3H): n = 181*, Nbw14^NZO/C3H^* (N_2_(NZOxC3H): n = 126, *Nbw14^NZO/NZO^* (N_2_(NZOx129P2): n = 133*, Nbw14^NZO/129P2^* (N_2_(NZOx129P2): n = 170; N, NZO.

In both crossbreedings, NZO-allele carriers for *Nbw14* gained more body weight compared to heterozygous allele carriers (N_2_(NZOxC3H): 62.4 g in *Nbw14*^NZO/NZO^ compared to 58.9 g in *Nbw14^NZO/C3H^* at 20 weeks of age, N_2_(NZOx129P2: 53.8 g in *Nbw14^NZO/NZO^* compared to 49.2 g in *Nbw14^NZO/129P2^* at 20 weeks of age; *P* < 0.001, Fig. 4A). These differences in body weight were attributed to significant differences in body fat (N_2_(NZOxC3H): 28.0 g in *Nbw14*^NZO/NZO^ compared to 25.4 g in *Nbw14^NZO/C3H^* at 15 weeks of age, N_2_(NZOx129P2): 22.7 g in *Nbw14^NZO/NZO^* compared to 20.3 g in *Nbw14^NZO/129P2^* at 15 weeks of age; *P* < 0.001, Fig. 4B). In contrast, the development of lean mass was similar between the different genotypes (Fig. 4C).

### Combined haplotype- and gene expression analysis identifies two candidate genes for *Nbw14*

**Figure 5:**
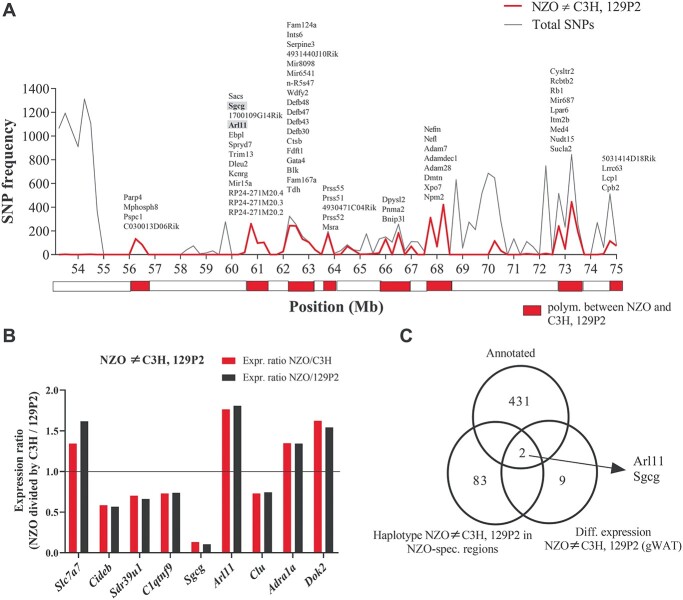
Combined approach of haplotype- and gene expression analysis in gWAT of the parental strains for the identification of NZO-specific gene variants within *Nbw14*. (**A**) Haplotype analysis using Genome Reference Consortium Mouse Build 38 (GRC38) provided by the Wellcome Trust Sanger Institute. The red line represents the number of polymorphic SNPs according to NZO ≠ 129P2 and C3H, whereas the grey line shows the total number of SNPs (all SNPs annotated for C57B6/J reference genome with calls for C3H, 129 and NZO). Both lines overlap in the regions between 56.5–56.75 Mb, 60.25–61.5 Mb and 68.0–68.5 Mb. Genomic regions containing less than 100 polymorphic SNPs (NZO ≠ 129P2 and C3H) per window (250 kb) are represented with the white boxes. In contrast, regions with more than 100 polymorphic SNPs (NZO ≠ 129P2 and C3H) per window are highlighted by red boxes. Genes located in regions that were defined as polymorphic between NZO and the other strains are listed above. For a better overview, gene models are not included. (**B**) Significantly (*P* < 0.05) differential gene expression in NZO vs. C3H and 129P2 at the age of 6 weeks in gWAT detected by microarray analysis. Higher expression in NZO is indicated with an expression ratio (NZO/C3H and NZO/129P2) > 1, whereas downregulation in NZO is shown with an expression ratio (NZO/C3H and NZO/129P2) < 1. Differences between the strains were calculated by one-sided Wilcoxon signed rank test. (**C**) Venn diagram showing the total number of annotated genes (top), the number of genes revealed from the haplotype analysis (left), the number of genes revealed from the microarray analysis (right) and the overlap of genes (centre).

Based on the assumption that the causal gene variant for *Nbw14* is different in NZO compared to C3H and 129P2 genomes, we used two major approaches to search for potential candidate genes. First, we used the SNP database from the Wellcome Trust Sanger Institute (28,29) to search for NZO polymorphisms in the defined critical region (53–75 Mb) that differed from C3H and 129P2. Since the genomic sequence for our C3H/FeJ substrain is not available in the database, the sequence from the closely related C3H/HeJ strain was used instead. As described before (11), for the dissection of the QTL peak into regions that are identical by decent (IBD) and polymorphic between NZO and the two lean strains, we determined the number of SNPs according to NZO ≠ C3H and 129P2 each 250 kbp. Regions exceeding a threshold of 100 SNPs/window were defined as polymorphic between NZO and C3H or 129P2, respectively (Fig. 5A). Out of 431 protein coding genes, only 83 were found to be located in regions defined as polymorphic between NZO and the other two strains. Supplementary Material, File S3 lists all annotated SNPs according to NZO ≠ C3H and 129P2 from all these 83 genes. We further analyzed these genes for coding nonsynonymous SNPs and found 42 protein polymorphisms where NZO differs from C3H and 129P2. According the ‘Sorting Tolerant From Intolerant’ (SIFT) algorithm (30), three variants in two genes (W277R in *Pnma2*, G285D and T330M in *Rubcnl*) are predicted to have deleterious impact on protein function (Supplementary Material, Table S2, Supplementary Material, File S2). However, none of these variants is unique for the NZO strain since these variants are shared by several lean mouse strains from the Sanger panel.

In addition, we implemented transcriptome data from gonadal white adipose tissue (gWAT) of our parental strains NZO, C3H and 129P2. In total, nine genes revealed to be either up- or downregulated in NZO gWAT compared to the other two strains. Whereas the expressions of *Slc7a7*, *Arl11*, *Adra1a* and *Dok2* were upregulated, the five genes *Cideb*, *Sdr39u1*, *C1qtnf9*, *Sgcg* and *Clu* showed lower mRNA expression levels in NZO compared to the other two strains (Fig. 5B). However, only two (*Arl11* and *Sgcg*) out of nine NZO-regulated genes were located in regions that were defined as highly polymorphic between NZO and the other strains. Thus, *Arl11* and *Sgcg*, the only two genes that overlapped between our candidate gene approaches (Fig. 5C) were selected for further analysis as our main candidate genes for *Nbw14*.

### *Sgcg* and *Arl11* expressions are NZO-specifically regulated exclusively in adipose tissue

**Figure 6:**
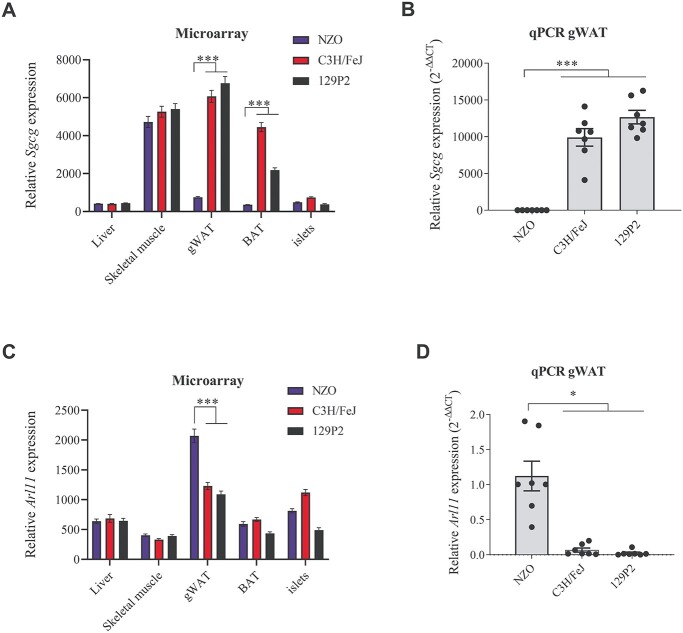
*Sgcg* and *Arl11* mRNA expressions across different tissues from the parental strains and validation in gWAT by qRT-PCR. All tissues were collected from male NZO, C3H and 129P2 mice at 6-weeks of age. The expressions of *Sgcg* and *Arl11* in liver, skeletal muscle, gWAT, BAT and pancreatic islets was measured by microarray analysis and the bars show the relative fluorescence units (**A** and **C**). Differential expression in gWAT tissue was confirmed by qRT-PCR (**B** and **D**). *Tbp* was used as endogenous control. Data represent mean values ± SEM from 5 (microarray) or 7 (qRT-PCR) mice each strain, respectively. Differences between the strains were calculated by two-sided Wilcoxon test (microarray) or One-way analysis of variance followed by post hoc Bonferroni test (qRT-PCR), ^*^P < 0.05, ^*^^*^^*^P < 0.001. gWAT, gonadal white adipose tissue; qPCR, qRT-PCR.

Using microarray data from several T2D-relevant tissues of our parental strains, we further compared the relative expression levels of *Arl11* and *Sgcg* between white adipose tissue, liver, skeletal muscle, brown adipose tissue and pancreatic islets. In C3H and 129P2, expression levels for *Sgcg* could be exclusively observed in skeletal muscle, brown- and white adipose tissue, whereas liver and pancreatic islets did not show any *Sgcg* expression. In contrast, in NZO, *Sgcg* expression was limited to skeletal muscle (Fig. 6A). *Arl11* revealed highest expression in gWAT tissue in all three strains. NZO-specific expression was observed exclusively in gWAT tissue, whereas no expression differences were detected in the other four tissues. In contrast to *Sgcg*, which was NZO specifically downregulated, *Arl11* revealed to be NZO specifically upregulated in adipose tissue (Fig. 6C). NZO-specific gene expressions for *Sgcg* and *Arl11* in gWAT were further validated by quantitative real-time polymerase chain reaction (qRT-PCR; Fig. 6B and D). Gene expression levels were markedly higher for *Sgcg* in the two lean strains (Ct values 23.0 in C3H, 23.2 in 129P2, 36.6 in NZO) compared to *Arl11* in the obese strain (Ct values: 30.0 in NZO, 34.6 in C3H and 36.7 in 129P2). In addition, the differences in gene expression were bigger for *Sgcg* (ΔCt: 13.3 and 13.2) than for *Arl11* (delta ΔCt: 6.7 and 4.6).

### *Sgcg* and *Arl11* expression QTL overlap with *Nbw14*

**Figure 7:**
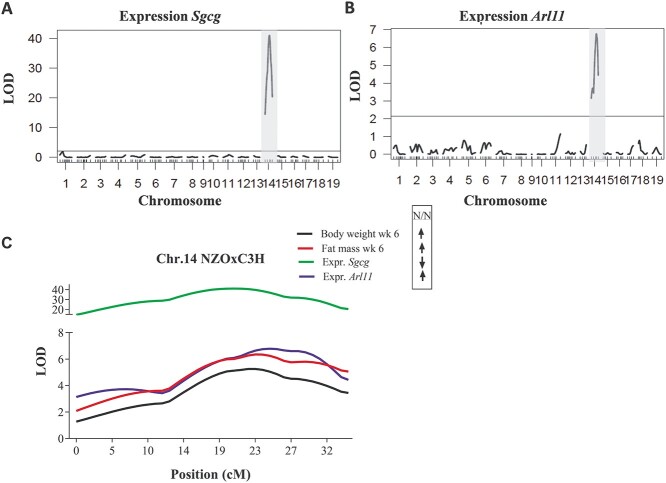
Expression QTL analysis for *Sgcg* and *Arl11* expressions in gWAT from all N_2_(NZOxC3H) mice. Genome-wide LOD scores distribution for the mRNA expressions of *Sgcg* (**A**) and *Arl11* (**B**) in N_2_(NZOxC3H) males, Chr.14 is highlighted in grey and the significance threshold (*P* < 0.05) is indicated with the horizontal line. (**C**) Expression QTL curves and metabolic QTL curves (body weight and fat mass) on Chr.14 were superimposed. The SNP-markers that were used for genotyping with the corresponding Mb-positions are plotted below. The genotypic effects are indicated with the arrows. All LOD scores were calculated with R/qtl package as described in methods. LOD, logarithm of the odds; Expr, expression; N, NZO; wk, week.

We further quantified the expression of *Sgcg* and *Arl11* in *gWAT* of each male N_2_(NZOxC3H) mouse and mapped the mRNA levels to the genome. The linkage analysis revealed *cis*-expression QTL (eQTL) on chromosome 14 for the expression of both genes, *Sgcg* (LOD 41.1 at 20.5 cM, closest SNP marker at 63.54 Mb, Fig. 7A) and *Arl11* (LOD 6.8 at 25 cM, closest SNP marker at 77.52 Mb, Fig. 7B). Both eQTL showed similar LOD score profiles as *Nbw14* (body weight: LOD 5.2 at 23 cM; fat mass LOD 6.3 at 23 cM; Fig. 7C).

### Overexpression of *Sgcg* increases glucose uptake in 3T3-L1 adipocytes

**Figure 8:**
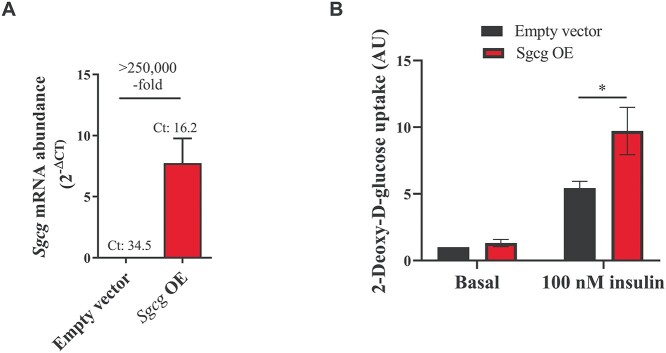
Measurement of 2-Deoxy-D-glucose uptake in *Sgcg* overexpressing 3T3-L1 vs. control adipocytes. Undifferentiated 3T3-L1 cells were infected with either a retrovirus carrying cDNA from *Sgcg* (*Sgcg* OE) or the empty pMSCV-puro vector as control and differentiated to adipocytes. The mRNA overexpression was confirmed by quantitative Realtime PCR (**A**). *Hprt* was used as endogenous control. 2-Deoxy-D-glucose uptake was determined in fully differentiated cells by measuring luminescence intensity at basal as well as after 60 min stimulation with 100 nM insulin (**B**). Bars represent mean values ± SEM from 3 (A) or 7 (B) different experiments. Statistical differences were calculated by two-way analysis of variance (B) followed by post hoc Bonferroni test, ^*^P < 0.05.

For functional analysis, we focused on the gene *Sgcg* as most likely candidate for *Nbw14*. Undifferentiated 3T3-L1 cells were infected with either a retrovirus carrying cDNA from *Sgcg* (*Sgcg* OE) or the empty vector and differentiated into adipocytes. The success of the overexpression was confirmed by determining *Sgcg* mRNA levels in *Sgcg* OE vs control adipocytes using qRT-PCR. The Ct-value dropped from 34.5 in control- to 16.2 in *Sgcg* OE cells (Fig. 8A). 2-Deoxy-d-glucose uptake was measured in fully differentiated cells at basal state as well as after exposure to 100 nM insulin for 60 min. While basal 2-deoxy-d-glucose uptake was not different, insulin-stimulated 2-deoxy-d-glucose uptake was significantly increased by approx. 60% in *Sgcg* OE compared to control cells (Fig. 8B). However, lipid accumulation obtained by Oil Red O staining was not altered in *Sgcg* OE cells (Supplementary Material, File S2, Supplementary Material, Fig. S1). We further measured AKT and pAKT protein abundances in SGCG overexpressing cells, but could not detect any differences compared to control cells (Supplementary Material, File S2, Supplementary Material, Fig. S2).

### *SGCG* expression correlates with body mass index and body fat in human fat tissue exclusively from T2D subjects

**Figure 9:**
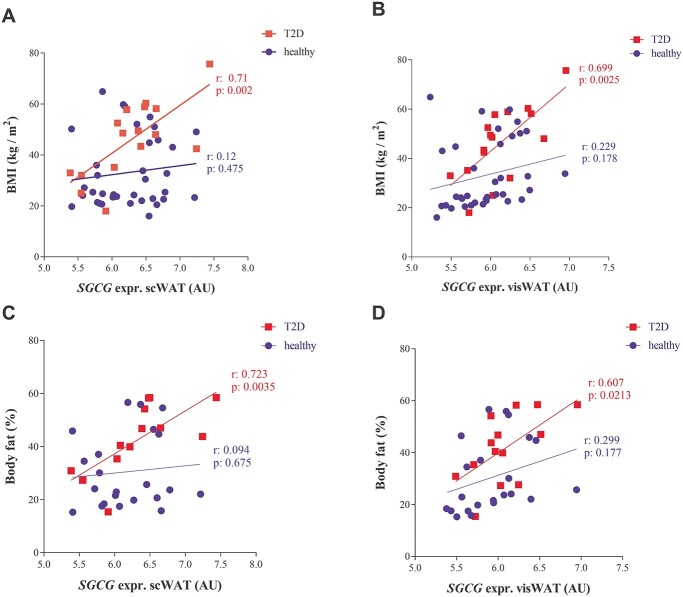
*SGCG* expression analysis in human adipose tissue. Correlation of *SGCG* mRNA levels with BMI in subcutaneous (**A**) and visceral adipose tissue (**B**) from healthy (blue circles) vs. patients with type 2 diabetes (T2D, red squares). Correlation of *SGCG* mRNA levels with body fat in subcutaneous (**C**) and visceral adipose tissue (**D**) from healthy controls (blue circles) vs. T2D (red squares) subjects. Correlations were calculated using Pearson correlation test. n = 16–36 for BMI and 14–22 for body fat.

To study whether *SGCG* may be also relevant for human obesity and T2D, we analyzed its expression in human subcutaneous and visceral adipose tissues from both patients with T2D and healthy controls. *SGCG* mRNA levels significantly correlated with body mass index (BMI) in subcutaneous (*r* = 0.71, *P* = 0.002; Fig. 9A) as well as visceral (*r* = 0.699, *P* = 0.0025; Fig. 9B) adipose tissue exclusively from diabetic, but not from healthy subjects (subcutaneous fat: *r* = 0.12, *P* = 0.475; visceral fat: *r* = 0.229, *P* = 0.178). A similar pattern was observed for the correlation with body fat. Whereas *SGCG* expression significantly correlated with body fat in subcutaneous (*r* = 0.723, *P* = 0.0035; Fig. 9C) and visceral (*r* = 0.607, *P* = 0.0213; Fig. 9D) adipose tissue from diabetic patients, no correlation was observed in healthy subjects (subcutaneous fat: *r* = 0.094, *P* = 0.675; visceral fat: *r* = 0.299, *P* = 0.177).

## Discussion

To identify novel obesity-modifier genes, we analyzed two backcross populations derived from diabetes-prone NZO mice and two lean, diabetes-protected strains, 129/P2 and C3H, respectively. Genome-wide linkage scans and transcriptome analysis of adipose tissue from mice identified *Sgcg* as a novel potential susceptibility gene for obesity. In human adipose tissue, *SGCG* expression significantly correlated with BMI and body fat exclusively in patients with T2D, suggesting that *SGCG* may represent a novel marker for diabetic obesity.

The NZO mouse strain represents a model for polygenetic obesity and T2D in humans. Both male and female mice develop obesity and severe insulin resistance. However, diabetes-related weight loss during the course of the disease may interfere with studies of obesity-related traits in male NZO mice (11,18). Nonetheless, female mice are protected from subsequent β-cell failure, which has been attributed to protective effects of oestrogen (16,17). In humans, the prevalence for T2D increases in women after menopause (31), also suggesting diabetes-protective effects of oestrogen.

Linkage analysis of female mice from two outcross populations, NZOxF_1_(129P2xNZO) and NZOxF_1_(C3HxNZO), revealed seven novel QTL for body weight and/or fat mass on six different chromosomes. Most of the QTL showed linkage exclusively in only one of the two backcross populations, suggesting that the underlying gene variants are derived from the respective lean strain, C3H or 129P2. In contrast, the locus *Nbw14* showed linkage to obesity-related traits in both backcross populations, indicating that the causal gene variant is likely specific for the NZO strain as NZO allele carriers in both crosses displayed elevated body weight and fat mass. Based on this assumption, we applied two major criteria to filter for candidate genes that may drive the high prevalence for obesity in NZO: NZO-specific gene expression in gWAT and location in haplotype blocks that are highly polymorphic between NZO and the two lean strains.

Because underlying genes for a QTL are likely located in genomic regions where the parental strains have different haplotypes (32,33), out of 431 annotated genes we exclusively considered genes as potential candidates for *Nbw14* that are residing in regions that are highly polymorphic between NZO and the other two strains*.* Our survey of the Sanger SNP database further revealed three missense mutations in the two genes *Pnma2* and *Rubcnl* in NZO that are likely to affect protein function. However, we found that these variants were also shared by several lean mouse strains listed in the Sanger database, indicating that these protein polymorphisms are not associated with an obesity phenotype as seen in NZO. Moreover, both genes showed only marginal expression in adipose tissue, defined as our main QTL-target tissue, from our mouse strains and from humans as observed in GTEx transcriptome database (34). However, both genes should not be excluded as candidates for *Nbw14*, since other tissues than adipose tissue, in particular skeletal muscle or the central nervous system (CNS), may be responsible for the phenotype mediated by *Nbw14*. *PNMA2* is highly expressed in the brain. The hypothalamus and other areas of the CNS have been established to play a key role in the regulation of energy homeostasis and food intake behaviour (35). In the future, it would be interesting to study a potential role of *PNMA2* in relation to food intake in the CNS.

Our genome-wide transcriptome analysis in gWAT revealed nine genes that either were up- or down regulated in NZO, compared to C3H and 129/P2. Out of these nine candidates, only two genes *Arl11* and *Sgcg* fulfilled our candidate gene criteria, thus representing the most likely candidates underlying the QTL.

Whereas *Arl11* revealed to be upregulated in gWAT from NZO, *Sgcg* expression was exclusively detected in the two lean strains. Interestingly, both genes showed NZO-specific expression exclusively in adipose tissue, whereas in other T2D-relevant tissues, such as skeletal muscle or liver, the mRNA expression was not different between our parental strains. This observation strengthens our hypothesis that both genes are plausible candidates for *Nbw14*, which likely interferes with adipose tissue function in obese NZO mice. In addition, eQTL analysis in gWAT from our N_2_(NZOxC3H) population revealed *cis*-signals for both genes within the *Nbw14* locus, thus providing evidence that both *Arl11* and *Sgcg* harbour a genetic sequence variation between NZO and C3H, presumably within the genes, that may be causal for *Nbw14*. The eQTL approach was already successfully used in the past as a tool to exclude and nominate genes for physiological QTL (36–39).

Due to the results from the gene expression analysis in gWAT from our mouse strains, which revealed clearly higher mRNA levels and expression differences for *Sgcg* than for *Arl11*, we decided to focus on *Sgcg* in this study. Nevertheless, a potential role of *Arl11* driving altered adipocyte function from NZO mice would need to be addressed in future studies. *Arl11* is known to be expressed exclusively in murine and human macrophages where it regulates their activation in response to LPS stimulation (40), indicating a role in the inflammatory response. In context to *Nbw14*, *Arl11* in pro-inflammatory adipose tissue macrophages from NZO mice may contribute to adipose tissue remodelling. It is broadly accepted that the amount of (pro-inflammatory) adipose tissue macrophages increases during obesity, leading to altered secretion profiles, surface marker expression and metabolic function of adipocytes, eventually contributing to the overall dysfunction of the adipose tissue (41). It remains to be investigated whether *Arl11* may take a causal role in this scenario or whether increased mRNA expression in NZO gWAT rather occurs secondarily as a consequence of obesity-induced inflammation.

*Sgcg* belongs to the Sacroglycan (SG) gene family, each of them encoding for a transmembrane glycoprotein. *Sgcg* is a vital component of the SG complex, a subcomplex of the dystrophin–glycoprotein complex, which maintains the integrity of the sarcolemma by linking the cytoskeleton and the extracellular matrix. Research on the SG proteins has been mainly limited to skeletal muscle in relation to muscular dystrophies (42). Recently it was shown that the dystrophin–glycoprotein complex interacts with the insulin receptor and regulates insulin signalling in skeletal muscle (43), suggesting that mutations in this complex contribute to the development of insulin resistance and T2D. Moreover, the disease Duchenne muscular dystrophy, which is caused by dissociation of the dystrophin glycoprotein complex, is associated with reduced insulin sensitivity in humans (44). However, the molecular basis of this interaction and the role of the SG complex remains to be elucidated.

In the present study, we found that retroviral overexpression of *Sgcg* in 3T3-L1 mouse adipocytes results in substantially increased insulin-mediated glucose uptake without affecting basal glucose uptake, suggesting a role of *Sgcg* in insulin action and energy metabolism. However, measurement of cellular lipid levels by Oil Red O staining failed to reveal any differences compared to control cells. More detailed studies are required to clarify whether deficiency in *Sgcg* may affect fatty acid uptake or de novo lipogenesis. Moreover, additional gene variants, including *Pnma2*, *Rubcnl* and *Arl11*, may also contribute to the phenotype mediated by *Nbw14*. We did not observe any changes in phosphorylated AKT (pAKT) levels between SGCG overexpressing vs. control cells, indicating that SGCG affects glucose transport downstream of AKT signalling, possibly at the level of GLUT4 vesicle trafficking. While the role of the SG complex in adipocytes remains unclear, β-sarcoglycan null mice, which lack the SG complex in adipose tissue and skeletal muscle, develop glucose-intolerance and whole body insulin resistance specifically due to altered insulin-stimulated glucose uptake in skeletal muscle (45). Further studies of *Sgcg* function in adipocytes are required to investigate this mechanism.

Finally, our expression analysis in human adipose tissue as well as different gWAS indicates that *SGCG* is also relevant for the pathogenesis of obesity and T2D in humans. Interestingly, we found a significant correlation of *SGCG* expression with BMI and body fat mass in human subcutaneous as well as visceral adipose tissue exclusively in T2D subjects, whereas no correlation was found in the healthy control group. This observation shows that *SGCG* can be used as a novel genetic marker for metabolically unhealthy obesity in humans. In line with our mouse data, which argue for a beneficial role of *Sgcg* on glucose homeostasis, we speculate that elevated levels of SGCG in diabetic humans may be compensatory to insulin resistance.

Interestingly, a common SNP (rs679482) that disrupts a functional enhancer intronic to *SGCG* has been associated with weight loss in response to energy restriction (46). Another SNP (rs9552911) in the human *SGCG* gene has been associated with T2D in a Chinese (47) as well as in a South Asian population (48). These findings indicate that there may also be a causal relationship between *SGCG* function and T2D pathogenesis in humans.

In conclusion, we again provide evidence that the comparative analysis of multiple inbred populations generated with one common breeding can substantially improve the mapping resolution of disease genes. Our strategy allowed the identification of *Sgcg* as a novel potential obesity-modifier gene in adipose tissue, which was successfully validated by our *in vitro* studies. Moreover, our translational approach shows that *Sgcg* represents a novel genetic marker for metabolically unhealthy obesity. Future studies are necessary to elucidate the mechanism by which *Sgcg* increases insulin-sensitivity in adipocytes. Nevertheless, since there is evidence that QTL regions may include multiple genes that contribute to the phenotype (49), it may be possible that further gene variants, including *Pnma2*, *Rubcnl* and *Arl11*, that may also act in other tissues, exert effects on regulatory circuits in the *Nbw14* locus. This may also include non-coding SNPs, indels, copy number polymorphisms, and yet unknown de novo mutations.

## Materials and Methods

### Animals and breeding strategy

All experiments were approved by the Ethics Committee (reference: 84–02.04.2013.A118) of the State Ministry of Agriculture, Nutrition and Forestry (State of North Rhine-Westphalia, Germany). Diabetes-prone NZO/Hl (NZO; German Diabetes Centre Duesseldorf) (50) and diabetes-resistant 129P2/OlaHsd (129P2; German Institute of Human Nutrition, Nuthetal, Germany) and C3HeB/FeJ (C3H; Helmholtz Center Munich, Germany) (51) mice were housed at three to six mice per cage (Macrolon type III) at a constant temperature of 22°C and a 12 h light–dark cycle (lights on at 6 a.m.). Animals had free access to food and water *ad libitum*. Female NZO and male 129P2 or C3H mice, respectively, were used to generate a F_1_ generation (NZOx129P2; NZOxC3H), and male F_1_ offspring was subsequently backcrossed with NZO females (N_2_: NZOxF_1_). For each backcross generation N_2_(NZOx F_1_(NZOxC3H)) and N_2_(NZOx F_1_(NZOx129P2)), approximately 300 females were generated, designated N_2_(NZOxC3H) and N_2_(NZOx129P2). After weaning at the age of 21 days, all experimental animals received a HFD containing 45 kcal% fat, 20 kcal% protein and 35 kcal% carbohydrates with 4.73 kcal/gm energy (D12451 Research Diets Inc., New Jersey, USA). At 21–22 weeks of age, mice were fasted for six hours before they were sacrificed by cardiac puncture under isoflurane anaesthesia. For the collection of gWAT for subsequent microarray analysis, NZO, C3H and 129P2 mice were sacrificed at 6 weeks of age.

### Genotyping

Genomic DNA was isolated from mouse tail tips using the InViSorb Genomic DNA Kit II (Invitek, Berlin, Germany). The genotyping was performed by KASP (Kompetitive Allele Specific PCR) using appropriate SNP assays (LGC genomics, Teddington, UK). Informative SNP markers (105 for N_2_(NZOx129P2) and 110 for N_2_(NZOxC3H)) (Supplementary Material, File S1) polymorph between NZO and 129P2 or C3H, respectively, were selected in a distance of 20 Mbp for each chromosome.

### Body weight and body composition

Body weight was determined at weeks 3, 6, 10, 15, 18 and 20 using an electronic scale. Body composition was measured at weeks 3, 6, 10 and 15 by noninvasive nuclear magnetic resonance spectroscopy (EchoMRI™-100 System, Echo Medical Systems, Houston, USA).

### Blood glucose levels

Blood glucose was measured at weeks 3, 6, 10, 15, 18 and 20 in the morning between 8 and 10 am using a CONTOUR® XT glucometer (Bayer Consumer Care AG, Leverkusen, Germany).

### RNA extraction and microarray analysis

Total RNA from gonadal adipose tissue (gWAT) (collected from 6-weeks old NZO, C3H and 129P2 mice, *n* = 5 per strain) and 3T3-L1 cells was isolated using the RNeasy mini kit (QIAGEN, Hilden, Germany) including DNAse digestion according to the manufacturer’s instructions. For microarray analysis of the gWAT, the quality of the isolated RNA was tested using an RNA 6000 nano kit (Agilent Technologies, Taufkirchen, Germany). Only samples with RIN values >8 were selected for subsequent microarray analysis. Genome wide expression analyses (*n* = 5 per genotype) were performed with 150 ng RNA using Affymetrix-Chip (GeneChip® Mouse Gene 1.0 ST Array) as previously described (11).

### cDNA synthesis and qRT-PCR

cDNA was synthesized using GoScript™ Reverse Transcriptase Kit (Promega, Madison, USA) using 500 ng RNA. For qRT-PCR, the GoTaq® qPCR Master Mix (Promega, Madison, USA) on a QuantStudio 7 Flex PCR System (Applied Biosystems, Foster City, USA) was used. *Tpb* was used as an endogenous control for gWAT from NZO, C3H and 129P2 mice; *Actb* for gWAT from all N_2_ mice; and *Hprt* for 3T3-L1 cells. Gene expression was quantified using the 2^−ΔΔCT^ method (52). For eQTL analysis, we used 2^-ΔCT^ expression values for linkage scans with R/qtl package of R as described below.

### Cell culture and differentiation of 3T3-L1 cells

3T3-L1 fibroblasts (Cl-173, ATCC, USA, negatively tested for mycoplasma contamination) were cultured in Dulbecco’s Modified Eagle Medium (Thermo Fisher Scientific) with 25 mM glucose, supplemented with 10% new-born calf serum and 1% penicillin/streptomycin. For differentiation into adipocytes, the fibroblast were grown to confluence and incubated with differentiation medium #1 (Dulbecco’s modified Eagle’s medium with 25 mM glucose, 10% fetal calf serum, 1% penicillin/streptomycin, 1 μg/ml insulin, 1 μM dexamethasone, 2 μM rosiglitazone and 0.5 μM IBMX. On day 7 of differentiation, differentiation medium #1 was replaced with differentiation medium #2 (medium #1 without dexamethasone, rosiglitazone and IBMX), and cells were cultured for additional 7 days until functional investigation.

### Retroviral overexpression

ORF sequence of the *Sgcg* cDNA clone (Clone ID: OMu09100C, NM_011892.3, GenScipt) was subcloned into the retroviral vector pMSCV-puro (Addgene plasmid #68469) using the XhoI-EcoRI restriction site. *Sgcg*-pMSCV-puro and empty pMSCV-puro plasmids were transfected to Plat-E packaging cells (kindly provided from Prof. Dr Stork from the University of Duesseldorf, Germany) using Lipofectamin 2000 (Thermo Fisher Scientific). 48 hours post transfection, culture supernatants were centrifuged for 4 min at 300 x g, 6 μg/ml polybrene was added, and the viruses were added to 3T3-L1 fibroblasts overnight. Drug selection by 4 μg/ml puromycin was initiated 24 hours after infection.

### Oil Red O staining

Retroviral transduced 3T3-L1 fibroblasts were seeded into collagen-coated 12-well plates (30 000 cells per well) and differentiated to adipocytes. Adipocytes were rinsed with PBS twice and fixed with 4% formalin in PBS for 60 min. The cells were washed with 60% isopropanol twice and dried before the addition of freshly diluted Oil Red O solution. Oil Red O was prepared by diluting a stock solution (0.5 g of Oil Red O; Sigma) in 100 mL of isopropanol with water (60:40 vol/vol), followed by filtration. Cells were stained for 20 min and then washed 4x with water before they were photographed. Finally, the Oil Red O dye was extracted by adding 100% isopropanol, the solution was transferred to 96-well plates and quantified at OD 500 nm.

### Glucose uptake

Retroviral transduced 3T3-L1 fibroblasts were seeded into collagen-coated 96-well plates (7500 cells per well) and differentiated into adipocytes. One day prior the assay, cells were starved for 24 hours with DMEM without serum and antibiotics. Cells were either left untreated or stimulated with 100 nM insulin for 1 hour at 37°C. Uptake of 2-deoxy-D-glucose in the adipocytes was measured using glucose uptake-GloTM assay kit (Promega, Madison, WI, USA) and luminescence intensity (Relative light unit, RLU) was quantified according to the manufacturer’s instructions.

### Expression analysis in humans

Paired samples of subcutaneous and omental visceral adipose tissue were obtained from 55 individuals (37 women, 18 men). The age ranged from 16 to 85 years and the BMI from 16 to 76 kg/m^2^. All adipose tissue samples were collected during laparoscopic abdominal surgery as described previously (53). Subsequently, adipose tissue biopsies were processed and gene expression was quantified as described previously (13). According to American Diabetes Association (ADA) criteria (54), the cohort was subdivided into patients with T2D and healthy normoglycemic controls.

### Linkage analysis

Distributions of phenotypic data were tested for normality by the use of the D’Agostino-Pearson omnibus test (GraphPad Software Inc., La Jolla, CA, USA). While body weight and body composition data sets were normally distributed, blood glucose data sets were log2-transformed to achieve normal distribution. QTL analyses including the genetic map, genotyping errors and linkage between individual traits and genotypes were performed on N_2_(NZOx129P2) (307 females) and N_2_(NZOxC3H) (310 females) populations using the R/qtl 1.40–8 package (55) of R (version i386 3.3.2). Single-QTL genome scans were performed by interval mapping with the Expectation–maximization (EM) algorithm (56). The significance thresholds (*P* < 0.05) for linkage were estimated by 1000 permutations (57). For the eQTL analysis, mRNA expression levels (2^-ΔCT^) from the N_2_(NZOxC3H) population were used as quantitative traits and mapped to the genome in a non-parametric linkage analysis as described above.

### Sequence and haplogroup analysis

Data for mouse SNPs and SNP-Gene assignments were from the Sanger Welcome Trust Institute Database (https://www.sanger.ac.uk/sanger/Mouse_SnpViewer). Coding nonsynonymous SNPs were analyzed for their potential impact on protein function using the ‘SIFT’ algorithm (http://sift.jcvi.org; (30). The chromosomal region was dissected into intervals of 250 kb to determine the frequency of polymorphic SNPs between the mouse strains as described previously (11). A window of 250 kb exceeding the threshold of 100 SNPs was defined as polymorphic according to the assumption NZO ≠ C3H, 129P2. For the determination of the total number, all SNPs annotated for the C57BL/6 J reference genome with calls for C3H/HeJ, 129P2/OlaHsd and NZO/HlLtJ were counted.

### Statistical analysis

Data are presented as mean values ± SEM. Statistical significance was reported by two-tailed Student’s t-test or one/two-way analysis of variance followed by *post hoc* Bonferroni test as appropriate. The Pearson correlation test was used to determine the relationship between *SGCG* expression and metabolic parameters in human subjects. Differences were considered significant when *P* < 0.05. Statistical analysis was conducted by GraphPad Prism 8 (GraphPad Software Inc., La Jolla, CA, USA).

## Data availability

Supplementary Material, File S1 contains R/qtl formatted mapping information, including mouse IDs, phenotype and gene expression data, and SNP marker identifiers, locations, and genotypes (A = NZO/NZO, H=NZO/C3H or NZO/129P2) from females of both N_2_(NZOxC3H and NZOx129P2) populations. Supplementary Material, File S2 contains further data supporting material. Supplementary Material, File S3 lists all annotated SNPs according to NZO ≠ C3H and 129P2 from all 83 genes located in regions that were defined as polymorphic between NZO and the other two strains. Microarray data are available under accession number GSE197101 (password: unqdeseynfcxnyf).

## Supplementary Material

File_S1_ddac150

File_S2_ddac150

File_S3_ddac150
