# Supplementary material for: Comparative genomic analyses of multiple backcross mouse populations suggest SGCG as a novel potential obesity-modifier gene
Source: Hum Mol Genet. 2022 Jul 7;31(23):4019–33. doi: 10.1093/hmg/ddac150 (PMC9703946; doi:10.1093/hmg/ddac150)
Supplement: File_S2_ddac150 [file file_s2_ddac150.docx]

**Supplementary materials**

**Comparative genomics of multiple backcross mouse populations identifies *SGCG* as a novel potential obesity-modifier gene**

Tanja Kuhn^1,2^, Katharina Kaiser^1,2^, Sandra Lebek^1,2^, Delsi Altenhofen^1,2^, Birgit Knebel^1,2^, Ralf Herwig^4^, Axel Rasche^4^, Angela Pelligra^1,2^, Sarah Görigk^1,2^, Jenny Minh-An Khuong^1,2^, Heike Vogel^2,3^, Annette Schürmann^2,3^, Matthias Blüher^5^, Alexandra Chadt^1,2^ and Hadi Al-Hasani^1,2§^

^1^Institute for Clinical Biochemistry and Pathobiochemistry, German Diabetes Center (DDZ),

Heinrich Heine University, Medical Faculty, Duesseldorf, D-40225, Germany

^2^German Center for Diabetes Research (DZD), Munich-Neuherberg, D-85764, Germany

^3^Department of Experimental Diabetology, German Institute of Human Nutrition Potsdam-

Rehbruecke, Nuthetal, D-14558, Germany

^4^Department of Computational Molecular Biology, Max Planck Institute for Molecular Genetics, Berlin, D-14195, Germany

^5^Helmholtz Institute for Metabolic, Obesity and Vascular Research (HI-MAG) of the Helmholtz Zentrum München at the University of Leipzig and University Hospital Leipzig, Leipzig, D-04103, Germany

^§^Corresponding author

**Table S1: Primers used for quantitative real-time PCR.** Primer sequences were synthesized by Eurogentec (Seraing, Belgium). Primers were dissolved in nuclease free water to reach a final concentration of 10 nM. Fwd, Forward; Rev, Reverse.

| **Target** | **Sequence 5'→3'** |
| --- | --- |
| *Actb* | Fwd: CCACCATGTACCCAGGCATT  Rev: AGGGTGTAAAACGCAGCTCA |
| *Arl11* | Fwd: GGGAGGATGTCTGCCCTAAC  Rev: CACTGAGCTCCTACCCTCTG |
| *Hprt* | Fwd: GGGCTTACCTCACTGCTTTC  Rev: TCTCCACCAATAACTTTTATGTCC |
| *Sgcg* | Fwd: CCCCAACTCGGAGTCTAAGC  Rev: ATCCAGCACCAGCACTCCTT |
| *Tbp* | Fwd: GCGGCACTGCCCATTTATTT  Rev: GGCGGAATGTATCTGGCACA |

**Table S2: Coding non-synonymous single nucleotide polymorphisms from genes in NZO-polymorphic haplotype blocks within the critical QTL region of *Nbw14*.** The Sanger database (REL-1505 - GRCm38; https://www.sanger.ac.uk) was queried for SNPs where NZO/HILtJ differed from both C3H/HeJ and 129P2/OlaHsd. SNPs were analyzed for their potential impact on protein function using the 'Sorting Tolerant From Intolerant' (SIFT) algorithm^1)^. Reference allele in bold face. As SIFT score below 0.05 predicts a deleterious impact of the substitution (rows highlighted in red).

| **Position Chr.14** | **Symbol** | **dbSNP** | **NZO** | **C3H** | **129**  **P** | **Protein positionn** | **Amino acid exchange** | **SIFT (score)** |
| --- | --- | --- | --- | --- | --- | --- | --- | --- |
| 56.674476 | *Mphosph8* | rs51133706 | A | **A** | T* | 319 | I⇒F | deleterious_low_confidence(0.03) |
| 61.173404 | *Sacs* | rs31485808 | G | **G** | A | 43 | G⇒S | tolerated_low_confidence(1) |
| 61.191245 | *Sacs* | rs50964652 | C | **C** | T | 251 | T⇒I | tolerated(0.25) |
| 63.017855 | *Defb43* | rs30487752 | A | **A** | G | 46 | I⇒V | tolerated(1) |
| 63.492747 | *Tdh* | rs30372412 | T | **T** | C* | 361 | N⇒S | tolerated(0.38) |
| 63.494406 | *Tdh* | rs30191872 | A | **A** | C* | 148 | V⇒G | deleterious_low_confidence(0.01) |
| 64.079295 | *Prss55* | rs30887883 | T | **T** | C* | 133 | T⇒A | tolerated(0.62) |
| 64.210466 | *Msra* | rs30425928 | A | **A** | T | 167 | L⇒M | tolerated(0.99) |
| 66.916319 | *Pnma2* | rs218806527 | C* | **C*** | T | 64 | A⇒V | tolerated(1) |
| 66.916336 | *Pnma2* | rs261717416 | G* | **G*** | A | 70 | V⇒M | tolerated(0.22) |
| 66.916895 | *Pnma2* | rs51829749 | A* | **A*** | G | 256 | K⇒R | tolerated(0.81) |
| 66.916957 | *Pnma2* | rs213604667 *^2)^* | T* | **T*** | C | 277 | W⇒R | deleterious(0.03) |
| 66.917145 | *Pnma2* | rs48457414 | G* | **G*** | C | 339 | E⇒D | tolerated(0.64) |
| 66.917150 | *Pnma2* | rs47442422 | G* | **G*** | A | 341 | C⇒Y | tolerated(0.18) |
| 68.120488 | *Nefm* | rs30628515 | A | **A** | G | 699 | L⇒P | tolerated_low_confidence(0.57) |
| 68.120700 | *Nefm* | rs31493517 | G | **G** | C | 628 | H⇒Q | tolerated(0.61) |
| 68.511273 | *Adam7* | rs50173050 | G* | **G*** | C | 508 | D⇒E | tolerated(1) |
| 68.511322 | *Adam7* | rs30102562 | T* | **T*** | C | 492 | K⇒R | tolerated(1) |
| 68571782 | *Adamdec1* | rs31027450 | C | **C** | G | 244 | V⇒L | tolerated(1) |
| 68.573170 | *Adamdec1* | rs49797727 | T | **T** | C | 179 | E⇒G | tolerated(1) |
| 68.607459 | *Adam28* | rs48702506 | C | **C** | T | 793 | R⇒K | deleterious_low_confidence(0) |
| 68.607460 | *Adam28* | rs51015410 | C | **C** | T | 793 | E⇒K | deleterious_low_confidence(0) |
| 68.607501 | *Adam28* | rs46017957 | A | **A** | G | 779 | I⇒T | tolerated_low_confidence(0.08) |
| 68.607518 | *Adam28* | rs47251533 | G | **G** | T | 773 | N⇒K | tolerated_low_confidence(0.24) |
| 68.609929 | *Adam28* | rs49319969 | G | **G** | A | 706 | P⇒S | tolerated(0.1) |
| 68.633158 | *Adam28* | rs30237700 | G | **G** | C | 355 | F⇒L | tolerated(0.65) |
| 68.637442 | *Adam28* | rs50839273 | G | **G** | C | 262 | A⇒G | tolerated(0.34) |
| 68.642038 | *Adam28* | rs47274101 | A | **A** | G | 187 | V⇒A | tolerated(0.48) |
| 68.649511 | *Adam28* | rs46458375 | C* | **C*** | A | 18 | G⇒V | tolerated(0.3) |
| 70.652367 | *Npm2* | rs50768380 | A* | **A*** | C | 36 | C⇒G | tolerated(0.05) |
| 70.696562 | *Xpo7* | rs31356904 | T | **T** | C | 251 | N⇒S | tolerated(0.25) |
| 73.661260 | *Gm21750* | rs579025520 | A | **A** | T | 12 | K⇒N | deleterious_low_confidence(0.02) |
| 73.661271 | *Gm21750* | rs582083288 | A | **A** | T | 16 | H⇒L | deleterious_low_confidence(0.01) |
| 75.032184 | *Rubcnl* | rs37259725 | G | **G** | A | 94 | S⇒N | tolerated(0.16) |
| 75.032213 | *Rubcnl* | rs259999767 | T | **T** | A | 104 | S⇒T | tolerated(0.16) |
| 75.036104 | *Rubcnl* | rs231759601 | A | **A** | G | 252 | D⇒G | tolerated(0.74) |
| 75.036145 | *Rubcnl* | rs247293487 | A | **A** | G | 266 | M⇒V | tolerated(1) |
| 75.036164 | *Rubcnl* | rs260796638 | T | **T** | C | 272 | I⇒T | tolerated(0.65) |
| 75.036203 | *Rubcnl* | rs229401080 *^3)^* | G | **G** | A | 285 | G⇒D | deleterious(0.02) |
| 75.038895 | *Rubcnl* | rs263265462 | A | **A** | C | 330 | T⇒P | tolerated(0.13) |
| 75.038896 | *Rubcnl* | rs36639039 *^4)^* | C | **C** | T | 330 | T⇒M | deleterious(0.04) |
| 75.260702 | *Cpb2* | rs46826569 | G | **G** | C | 102 | E⇒Q | tolerated(0.15) |

^1)^ Kumar, P., S. Henikoff and P. C. Ng, 2009 Predicting the effects of coding non-synonymous variants on protein function using the SIFT algorithm. Nat Protoc 4: 1073-1081. ^2)^ 25 out of 37 strains from the Sanger database share C allele (W⇒ R) for rs213604667. ^3)^ 4 out of 37 strains from the Sanger database share A allele (G⇒ D) for rs229401080: MOLF/EiJ, NOD/ShiLtJ, NZO/HlLtJ, PWK/PhJ. ^4)^ 4 out of 37 strains from the Sanger database share T allele (T⇒ M) for rs36639039: MOLF/EiJ, NOD/ShiLtJ, NZO/HlLtJ, PWK/PhJ.

**
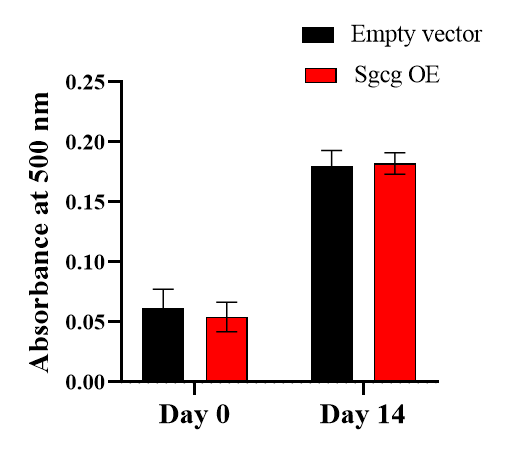
**

**Figure S1: Measurement of lipid accumulation in *Sgcg* overexpressing 3T3-L1 vs. control adipocytes.** Undifferentiated 3T3-L1 cells were infected with either a retrovirus carrying cDNA from *Sgcg* (*Sgcg* OE) or the empty pMSCV-puro vector as control. Lipid accumulation was measured by Oil-Red-O staining before differentiation (Day 0) and in fully differentiated adipocytes (Day 14). Bars represent mean values ± SEM from nine different experiments. Statistical differences were calculated by 2-way ANOVA followed by post hoc Bonferroni test.

**
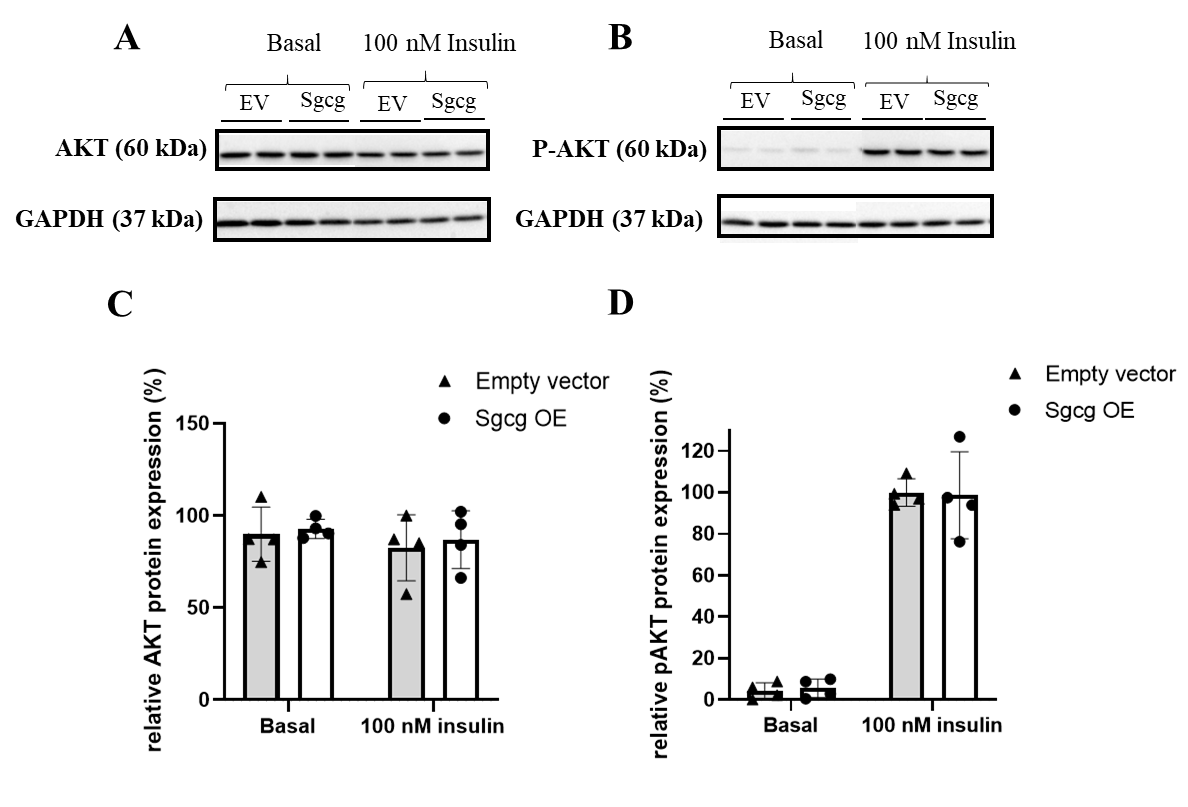
**

**Figure S2: Western blot analysis of the expression of AKT and phoyphorylated AKT in *Sgcg* overexpressing 3T3-L1 vs. control adipocytes.** Undifferentiated 3T3-L1 cells were infected with either a retrovirus carrying cDNA from *Sgcg* (*Sgcg* OE) or the empty pMSCV-puro vector as control and differentiated into adipocytes. Cells were harvested before (basal) and after stimulation with 100 nM insulin for 60 min. Representative Western blot membranes from *Sgcg* overexpressing and control adipocytes after analysis of AKT (**A**) and phoyporylated AKT (pAKT, **B**). Quantification of protein abundance for AKT (**C**) and pAKT (**D**). Data are presented as mean ± SEM from four different experiments. Empty vector vs. *Sgcg* OE (unpaired two-tailed Student t test).
